# Designing novel inhibitors against Mycobacterium tuberculosis FadA5 (acetyl-CoA acetyltransferase) by virtual screening of known anti-tuberculosis (bioactive) compounds

**DOI:** 10.6026/97320630014327

**Published:** 2018-06-30

**Authors:** Atul Kumar Jaiswal, Syed Hussain Abbas Husaini, Amarjeet Kumar, Naidu Subbarao

**Affiliations:** 1 School of Computational and Integrative Sciences, Jawaharlal Nehru University, New Delhi, India; 2 Department of Bio-Engineering, Integral University, Lucknow, Uttar Pradesh, India

**Keywords:** Inhibitors, Mycobacterium tuberculosis, FadA5, virtual screening, molecular docking

## Abstract

By-products of fatty acid degradation are extensively utilized by Mycobacterium tuberculosis (Mtb) for lipid synthesis and energy production during the infection phase. Cholesterol from host is scavenged by Mtb to fulfill its metabolic requirements, evade host immunity and invade macrophages. Blocking cholesterol catabolic pathways leads to bacteriostasis. FadA5 (Acetyl-CoA acetyltransferase), a thiolase encoded by fadA5 (Rv3546) gene in Mtb, plays a crucial role in cholesterol aliphatic chain degradation. Hence, FadA5 is a potential target for designing antitubercular inhibitors. In this study, 60,284 anti-tuberculosis (bioactive) compounds from ChEMBL database and analogous library from ZINC database of commercially available compounds have been screened against FadA5 active site to identify compounds having inhibitory potential against both the apo (state I) and the intermediate (state II) states of FadA5. Altogether, this study reports 7 potential inhibitors against two functional states of FadA5, which can be further taken for invitro studies.

## Background

Hypercholesterolemia is known to impair immunity against TB and contributes towards the infection development [1]. Host lipids have been shown to play an important role in Mtb survival against the host immunity [2]. Mtb primarily uses fatty acids as their main source of carbon during infection [3] and requires cholesterol for macrophage invasion [4]. Mtb does not synthesize cholesterol instead utilizes host cholesterol to accomplish its metabolism [5]. They penetrate the macrophage membrane where cholesterol-rich microdomains are present [6]. By-products of cholesterol catabolism are used by the bacterium for lipid synthesis and energy production [7]. Deletion of mce4 transporter in Mtb blocked cholesterol import thus resulting in reduced infection both in the activated and the mouse model [7]. Blocking cholesterol catabolic pathways at certain steps have been shown to cause bacteriostasis and cell deaths in Mtb [8]. These literature evidences justify that cholesterol and cholesterol catabolic pathway is crucial for Mtb survival in macrophages and can be targeted to develop new antitubercular drugs.

Cholesterol catabolism in Mtb starts with the degradation of aliphatic side chains of cholesterol followed by the sequential degradation of rings [5]. Many enzymes are involved in this process. FadA5 is one such enzyme, encoded by fadA5 (Rv3546) that catalyzes the thiolysis of keto CoA-esters formed during beta-oxidation of the cholesterol side chain [10] and produces androsterone metabolites, which contribute towards Mtb persistence [9]. The complete degradation process follows a pingpong mechanism where an acylated-cysteine intermediate is formed. This active site cysteine is crucial for catalysis as its mutation leads to attenuation of infection in the mouse model [9]. Recently, four structure variants of FadA5 have been published representing its apo form [PDB ID: 4UBW], wild-type acetyl-CoA bound form (WT-(Ac-)CoA) [PDB ID: 4UBV], CoA bound intermediate form (C93S-CoA Complex) [4UBU] and OPC (which is a small molecule steroid [10]) bound intermediate form, i.e., C93S OPC Complex [PDB ID: 4UBT].

In this study, we have screened known antituberculosis bioactive chemical library (ChEMBL database) and an analogous library (ZINC database) to identify novel active inhibitors against FadA5. The intermediate form of FadA5 (state II) has acetylated serine (OAS93), which is larger in size as compared to C93 present in FadA5 state I. This is why, firstly, CoA bound intermediate form (OAS93-CoA; acetylated-serine-CoA) was used to identify top hits which were then cross-docked against apo-form to identify potential inhibitors against both the functional states of the enzyme. We report 7 compounds, which have good predicted inhibitory potential towards state I and state II of FadA5.

## Methodology

**Figure 1:**
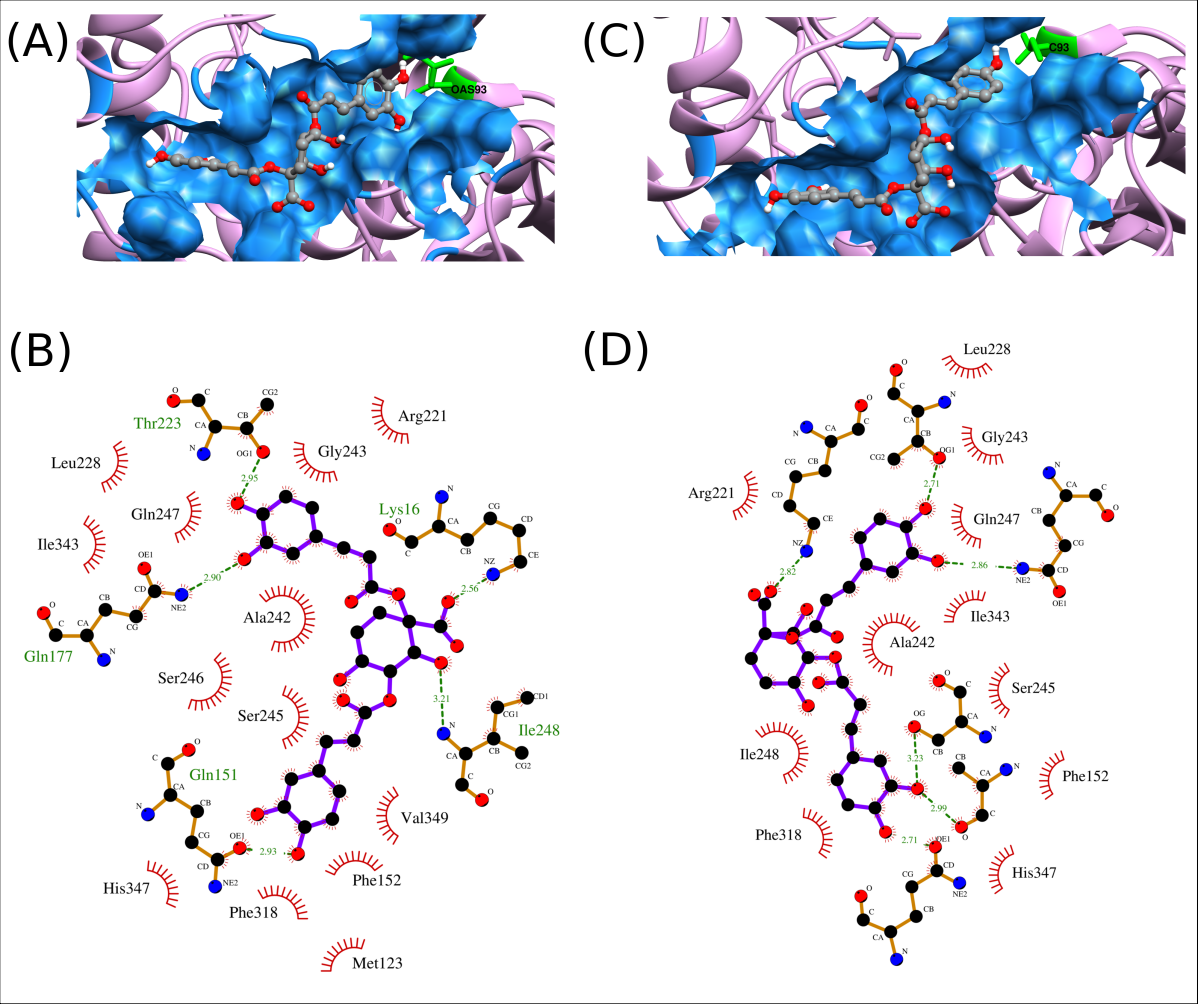
(A) FadA5 state II and Z1 complex, (B) FadA5 state II and Z1 interaction profile, (C) FadA5 state I and Z1 complex and (D) FadA5 state I and Z1 interaction profile. In (B) and (D), Z1 is represented in violet colored bonds, green color dashed line represents hydrogen bonds between Z1 and protein, red radial spokes represents residues forming hydrophobic interactions and hydrogen bond forming residues are represented in golden color bonds. Hydrogen bond lengths are labeled in Å.

The workflow used in this work is shown in Figure 1 (see supplementary material).

### Target protein structure

FadA5 belongs to thiolase family of enzyme, involved in the catabolism of fatty acids. The crystal structure of the enzyme was solved with acetyl-CoA and CoA. The active site of the enzyme consists of amino acid residues C93, R221, H347, A242, G243, Q177, S246, T224 and G227. In addition to active site residues, CoA, which is a substrate to the enzyme, also makes direct interactions with Q151, T223, and S246 and water-mediated interaction with Q247 [10]. In this study, two structures, one representing the apo form (PDB ID: 4UBW) and one representing the modified C93S variant of the 3-ketoacyl-CoA thiolase FadA5 (PDB ID: 4UBU) were selected as target, which will be referred as FadA5 state I and FadA5 state II, respectively in rest of the manuscript. FadA5 state II was obtained by modifying C93to OAS93 (acetylated-serine). Both the states of the protein were prepared using protein prep wizard of Schrodinger suite [11]. While preparation of the receptor, all water molecules were deleted and missing hydrogen atoms were added. Missing side chain residues were modeled using prime tool available in Schrodinger suite [12]. It was followed by restrained energy minimization by fixing the main chain to remove steric clashes between side chains. While preparation of the receptor of FadA5 state II, CoA was retained to define the active site of the enzyme. The receptor grid was generated using Glide module [13, 14] of Schrodinger suite. The receptor grid was generated using the center of CoA as grid center, and the grid boundary was defined in such a way that the minimum distance between any atom of ligand and grid boundary is at least 5Å. The active site of the apo form of FadA5 (state I) was defined by the amino acid residues, which are in close proximity of CoA in state II of FadA5, which was later, used for receptor grid generation.

### Selection and preparation of Chemical library

In the present work, two chemical libraries, viz ChEMBL antituberculosis (bioactive) database and ZINC analog were employed to identify inhibitors against FadA5. The ChEMBL database is publicly available [15] and consists of 2,101,843 compounds. From ChEMBL database, only the Mtb specific bioactive compounds (60284) library was selected for screening purpose. The ZINC database [16] contains 35 million purchasable compounds. From the ZINC database, only those chemicals were selected which were analogous to the top hits obtained from ChEMBL database (similarity cutoff >= 90%). Preparation of each chemical library was done in the following manner. First, small fragments were removed, then hydrogen atoms were added and finally, the structures were optimized. These steps were performed using Corina software [17]. And at last, the library was prepared using LigPrep module of Schrodinger Suite. The ionization state of the ligands was predicted using Epik [18] (pH, 7±2) and a maximum of 32 stereoisomers were generated.

### Screening of anti-tuberculosis (bioactive) compounds and its analogues against FadA5 state II

At first, the compounds from the prepared ChEMBL compound library were screened against the receptor grid generated for FadA5 state II. Starting with a total of 139498 prepared structures, first, virtual screening was performed in SP (Standard precision) mode [14] followed by XP (Extra Precision) [19] of top 10% hits, i.e., 13949 structures. In the later stage, top 10 ranked compounds based on G-Score obtained from XP in glide, were taken for further analysis. For docking in SP and XP mode, the upper limit for the allowed number of atoms in each ligand structure was kept to 200 and the upper limit for the allowed number of rotatable bonds was kept to 50. Additionally, epik state penalties [18] were added in calculation of scores in each case. In the last stage, screening of analogs of the top-ranked ChEMBL compounds from ZINC database was performed. The analogous library was screened directly in XP mode. The binding affinities of the obtained best binding pose of top ranking compounds were predicted using X-score (v1.2.1). X-score estimates the binding affinity of a given binding mode of a ligand within the binding pocket using empirical scoring function. Its scoring function estimates the binding affinity on the basis of four energy terms viz Van der Waals interaction energy, hydrogen bonding energy, deformation penalty and hydrophobic effect.

### Screening of top ranked compounds of FadA5 state II against FadA5 state I

The top-ranked compounds obtained after screening of ChEMBL and analogues library against FadA5 state II were re-docked against FadA5 state I in XP mode to identify compounds that bind to both the states of the enzyme. The compounds were then scored and evaluated.

## Results and Discussion

### Screening of anti-tuberculosis (bioactive) compounds against FadA5 State II

After screening ChEMBL compounds against FadA5 state II, the compounds were ranked on the basis of G-score. The G-Score of the top 10 compounds ranged from -12.252 kcal/mol (C1) to -10 34 kcal/mol (C10) Table 1 (see supplementary material). The predicted binding affinity of each compound using X-score lie in the range of -10.67 kcal/mol (C8) to -8.46 kcal/mol (C3) Table 1 (see supplementary material). To understand the interactions of top-ranked compounds, the 2D interaction profiles were generated using LigPlus, and different types of interactions between ligands and protein were analyzed.

As mentioned in Table 1 (see supplementary material), compound C8 possess the highest binding affinity towards FadA5 state II among all the top 10 compounds when examined in terms of X-score. Compounds C1, C2, C4, C5, C6, C7 and C10 were found to have binding affinities close to -9 kcal/mol calculated using X-score. Compound C3 although raked better than C4, C5, C6, C7, C8, C9 and C10in terms of G-score; the calculated binding affinity using X-score suggests that it has lower binding affinity compared to other compounds enlisted in top 10 list. All the top 10 listed compounds have more or less similar binding affinity towardsFadA5 state II as they share similar structure scaffold.

Combining all these properties, we can say that the compound C1 (2D interaction profile of C1 is shown in Figure 2 (see supplementary material) and C8 (2D interaction profile is shown in Figure 3 (see supplementary material)) have the highest inhibitory potential in terms of X-score, G-score, and the number of hydrogen bonds they share with binding pocket residues Figure 2 (see supplementary material).

### Molecular docking of analogues chemical library against FadA5 State II:

The docking studies for analogues compound library obtained from ZINC database was performed and the compounds were ranked on the basis of G-score Table 1 (see supplementary material). The Gscore and X-score of the top ten compounds (Z1-Z10) vary between -12.626 Kcal/mol (Z1) to -11.582 kcal/mol (Z10) and - 9.81 kcal/mol (Z2 and Z4) to -8.17 kcal/mol (Z10) Table 1 (see supplementary material), respectively. Compounds Z2 and Z4 were found to have the highest affinity, i.e., -9.81 kcal/mol; and compound Z10 was found to have the least affinity, i.e., -8.17 kcal/mol towards FadA5 state II as per X-score. 2D interaction profile of the complexes reveal that compounds Z2 and Z5 form the highest number of hydrogen bond interaction which involves amino acid residues Lys16, Arg 17, Gln151, Gln177, Thr223, Ser246 Ile248, Ala317, and Ile343, out of which the hydrogen bonds with all residues except Ile248 and Ser246 are conserved. The obtained results imply that compounds Z2 Figure 4 (see supplementary material) and Z5 Figure 3 (see supplementary material) have good affinity towards FadA5 state II.

### Molecular docking of anti-tuberculosis (bioactive, C1-C10) and analogues (Z1-Z10) compounds against FadA5 State I:

The FadA5 state I and state II represents the apo and intermediate forms of FadA5 and differs only in terms of one residue, i.e., in state II C93 is modified to acetylated-serine, which represents an intermediate functional state of the enzyme. Since, we were interested in identifying inhibitors against both the functional states of the enzyme FadA5, we cross-docked the topranked compounds from ChEMBL as well as ZINC library, which were found to have good binding affinity towards FadA5 state II, against FadA5 state I. The top-ranked compounds are listed in Table 1. The compounds, in this case, are referred with the same CID as with the FadA5 state II with a suffix "state I" to represent their interactions with FadA5 state I.

In case of FadA5 state I, the G-score ranges between -11.065 kcal/mol to -9.906 kcal/mol being lowest for Z1-state I and highest for C1-state I. The binding affinities predicted using Xscore ranges from -9.58 kcal/mol to -8.28 kcal/mol (Table 1). Z1- state I is shown to have the highest affinity towards FadA5 state I. The 2D interaction profile of Z1-state I with both the states of FadA5 is shown in Figure 1. For other compounds, the ranking order has changed, but have almost similar type of binding affinities with the FadA5 state I as have been observed for FadA5 state II. Number of hydrogen bond interaction ranges between 4 to 6, numbers of lipophilic interactions varies between 7-13 and number of non-bonded interactions varies between 42 and 63 (Table 1). These results imply that the compounds enlisted in Table 1 have good affinity towards both the functional states of FadA5.

Comparing the results obtained for various compounds after docking to FadA5 state II and FadA5 state I, it can be implied that all the top-ranked ChEMBL compounds enlisted in Table 1 and their analogs enlisted in Table 1 (see supplementary material), interact in the same fashion to both the states of FadA5. For example, Compound Z1, which is shown to have more affinity towards FadA5 state II Table 1 (see supplementary material) also has good binding affinity towards FadA5 state I (Table 1). From 2D plots of the complexes of Z1 with FadA5 state II and FadA5 state I as shown in Figure 1, it is quite obvious that this compound interacts with FadA5 state I and state II in a similar fashion and binds in same orientation forming same hydrogen bond interactions and have similar binding affinity towards both the states of the enzyme.

## Conclusion

Docking and post-docking analysis suggest that the top-ranked compounds reported in this study have similar type of interaction profile and affinity towards both the states of FadA5, except compound C3 and C9 which have relatively less affinity towards the enzyme. Altogether, this study reports 7 potential inhibitors against both the functional states of FadA5, which can be taken further for in-vitro studies.

## Supplementary material

Data 1

## Figures and Tables

**Table 1:** Docking and post docking results of analogues and ChEMBL chemical library against FadA5 state I (apo structure).

CID	Compound id from source database	G-Score (kcal/mol)	X-score (Kcal/Mol)	Number of HB	Number of Hydrophobic interactions	NIB
Z1-state I	ZINC86864386	-11.065	-9.58	6; Lys16, Gln151, Ala317, Ser246, Gln177, Thr223	11	56
Z8-state I	ZINC67913793	-11.006	-9.57	5; Lys16, Arg17, Gln151, Gln177, Thr223	8	62
Z4-state I	ZINC03919243	-10.54	-9.37	5; Lys16, Gln151, Ala317, Gln177, Thr223	13	62
Z7-state I	ZINC38143877	-10.346	-9.49	5; Arg17, Gln151, Ile343, Gln177, Thr223	11	63
C9-state I	CHEMBL233434	-10.102	-8.28	4; Arg17, Gln177, Ile248, Ser246	10	59
Z2-state I	ZINC39351841	-9.987	-9.2	5; Arg17, Ser246, Gln151, Ile343, Gln177	7	42
C1-state I	CHEMBL296650	-9.906	-9.36	6; Lys16, Ser246, Ala317, Gln177, Ile343, Thr223	11	49
CID: Compound identification number used in this paper. HB: hydrogen bond forming residues. NBI: Non-bonded interactions.
