# Supplementary material for: Designing novel inhibitors against Mycobacterium tuberculosis FadA5 (acetyl-CoA acetyltransferase) by virtual screening of known anti-tuberculosis (bioactive) compounds
Source: Bioinformation. 2018 Jun 30;14(6):327–36. doi: 10.6026/97320630014327 (PMC6137569; doi:10.6026/97320630014327)
Supplement: Data 1 [file 97320630014327S1.pdf]

BIOINFORMATION
Discovery at the interface of physical and biological sciences

Open access

Supplementary data:

Supplementary Figure 1: Docking workflow used in the present study.

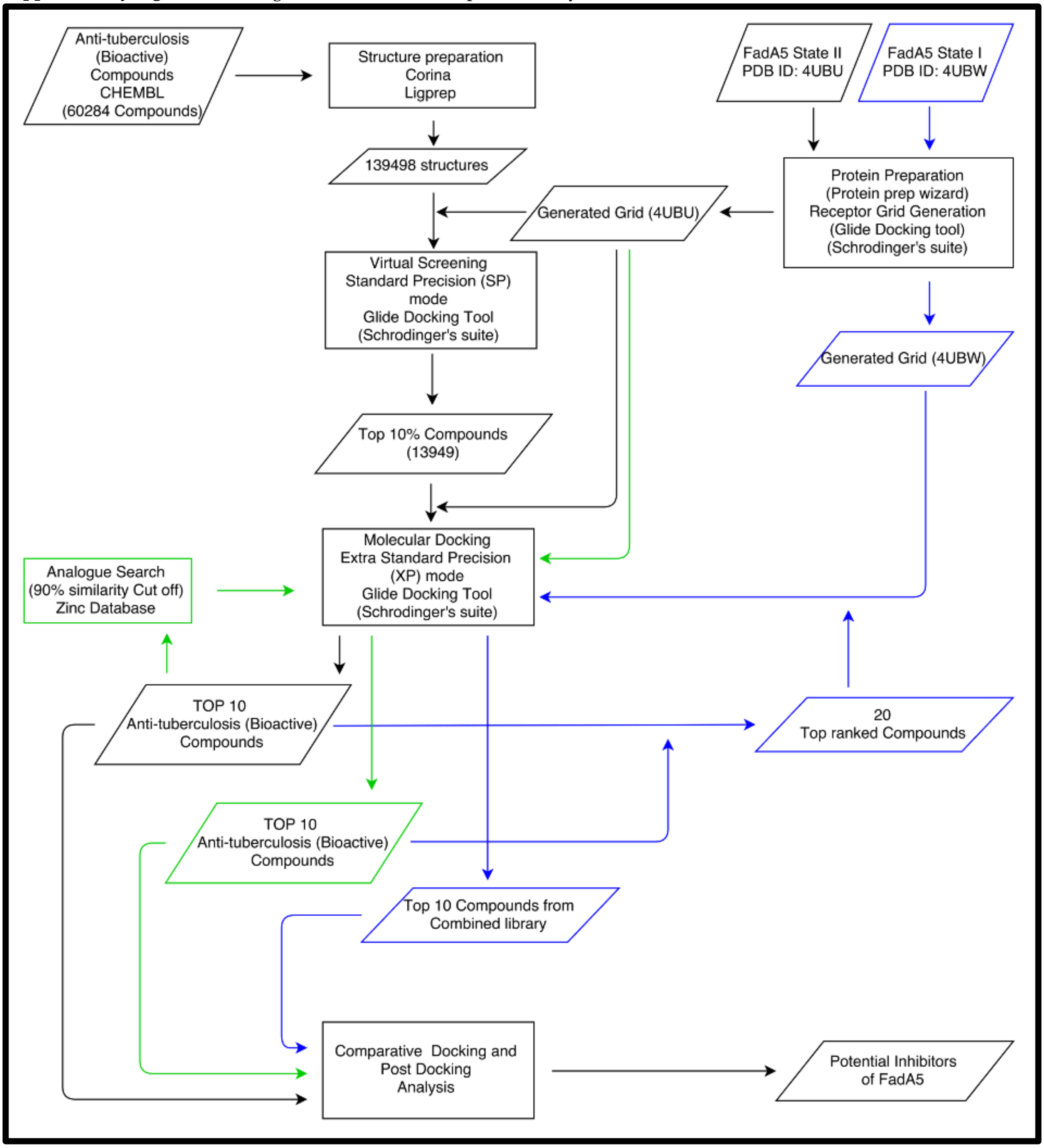

BIOINFORMATION
Discovery at the interface of physical and biological sciences

Open access

Supplementary Figure 2: (A) Compound C1 bound to FadA5 state II, (B) hydrogen bonding interaction of C1 and active site amino acid residues and (C) ligand interaction profile of C1 in the binding pocket. Legends of protein ligand interactions are given in **Figure 1.**

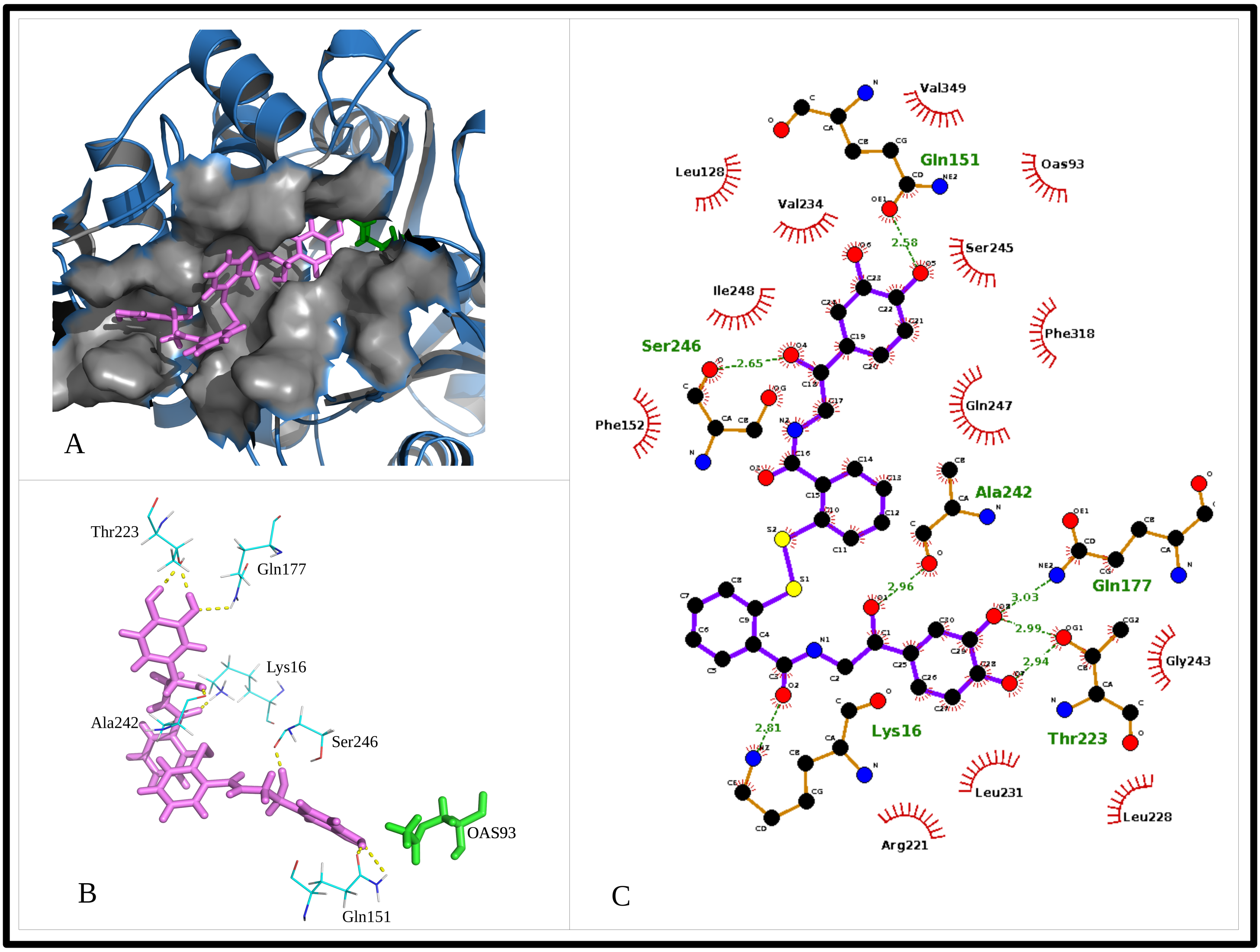

BIOINFORMATION
Discovery at the interface of physical and biological sciences

Open access

Supplementary Figure 3: (A) Compound C8 bound to FadA5 state II, (B) 2D interaction profile of C8 in the binding pocket, (C) Compound Z5 bound to FadA5 state II and (D) 2D interaction profile of Z5 in the binding pocket. Legends of protein ligand interactions are given in **Figure 1**.

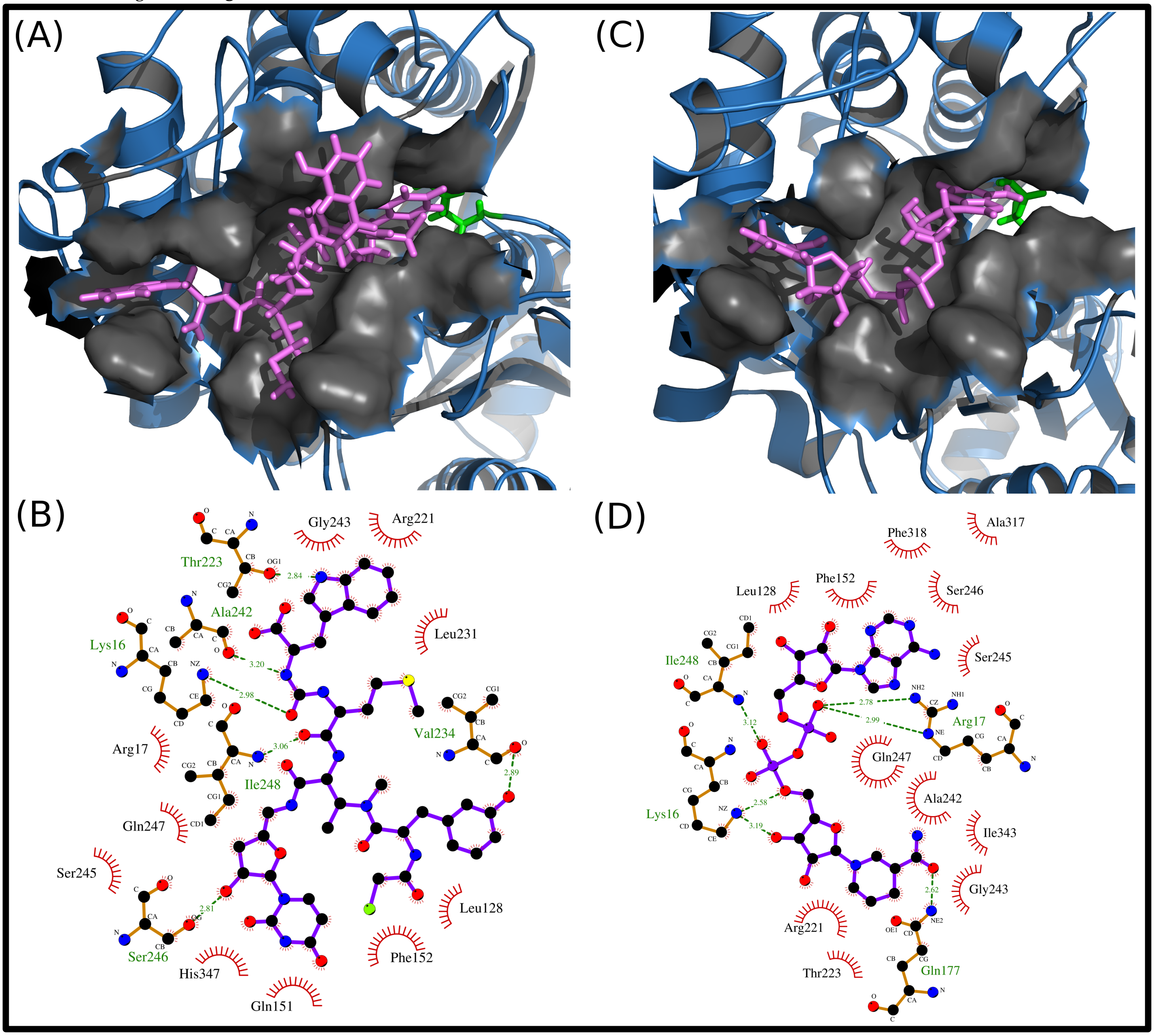

BIOINFORMATION
Discovery at the interface of physical and biological sciences

Open access

Supplementary Figure 4: (A) Compound Z2 bound to FadA5 state II, (B) hydrogen bonding interaction of Z2 and active site amino acid residues and (C) ligand interaction profile of Z2 in the binding pocket. Legends of protein ligand interactions are given in **Figure 1.**

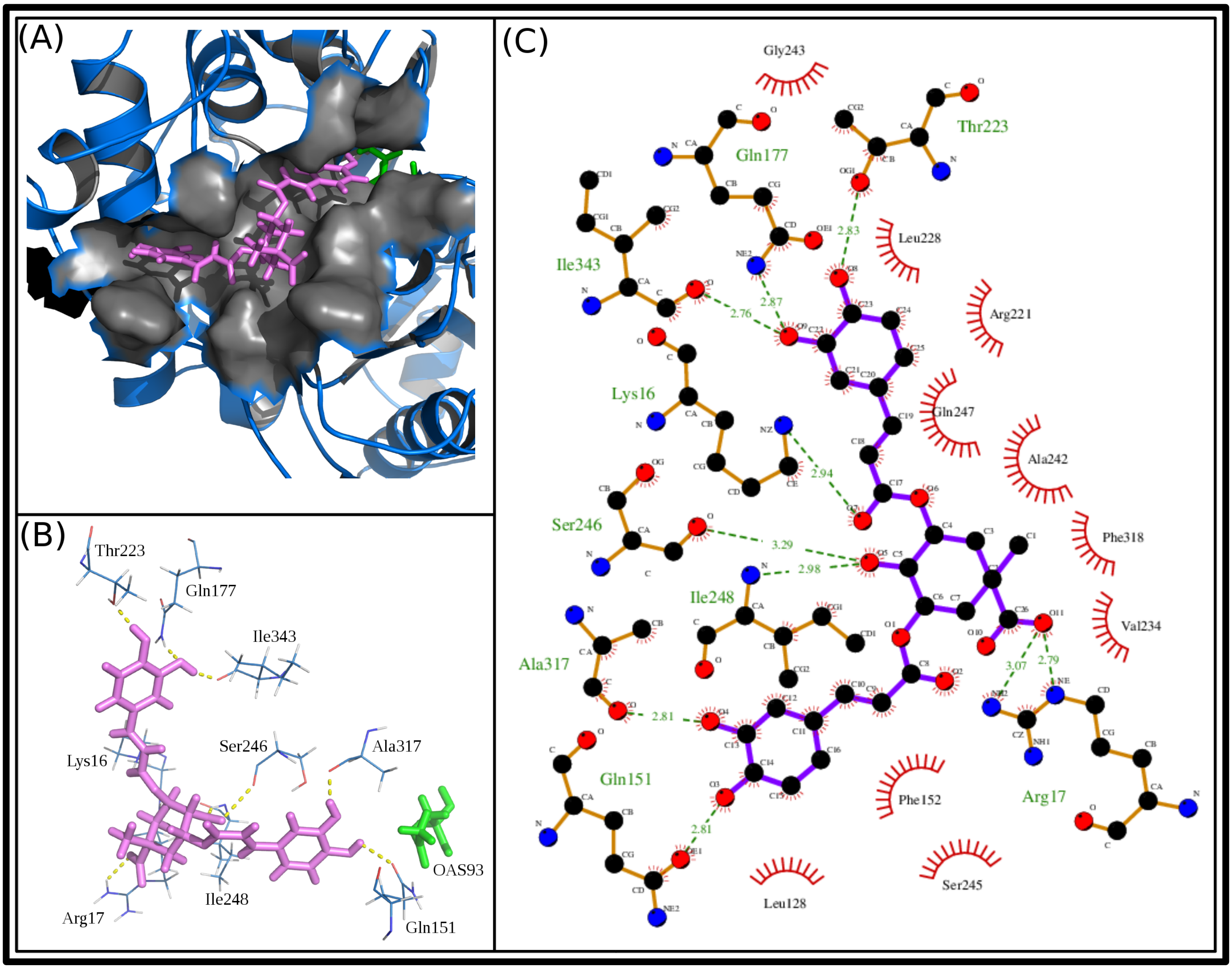

BIOINFORMATION
Discovery at the interface of physical and biological sciences

Open access

Supplementary Table 1: Top ranking compounds against FadA5 state II structure from virtual screening of known anti-tuberculosis compounds (ChEMBL bio-assay) and analogoues compounds (ZINC database).

FadA5 state II - ChEMBL				FadA5 state II - ZINC			
CID	IUPAC NAME (CHEMBL ID)	G-Score (Kcal/mol)	X-score (Kcal/Mol)	CID	IUPAC NAME (ZINCdatabase ID)	G-Score (Kcal/mol)	X-score (Kcal/Mol)
C1	(CHEMBL296650)	-12.252	-9.92	Z1	(ZINC86864386)	-12.626	-9.44
C2	(CHEMBL2024335)	-12.224	-9.10	Z2	(ZINC39351841)	-12.483	-9.81
C3	(CHEMBL250087)	-11.409	-8.46	Z3	(ZINC60392740)	-12.325	-8.46
C4	(CHEMBL2024340)	-11.405	-9.13	Z4	(ZINC03919243)	-12.198	-9.81
C5	(CHEMBL82570)	-11.065	-9.39	Z5	(ZINC60392741)	-12.012	-8.46
C6	(CHEMBL392204)	-10.957	-9.06	Z6	(ZINC77311642)	-11.817	-8.66
C7	(CHEMBL510826)	-10.867	-9.67	Z7	(ZINC38143877)	-11.732	-9.28
C8	(CHEMBL1910811)	-10.449	-10.67	Z8	(ZINC67913793)	-11.621	-9.45
C9	(CHEMBL233434)	-10.381	-8.70	Z9	(ZINC86864389)	-11.599	-9.34
C10	(CHEMBL247657)	-10.34	-10.40	Z10	(ZINC85480044)	-11.582	-8.17

CID: Compound identification number used in this paper.
